# Vitamin D Promotes Mucosal Barrier System of Fish Skin Infected with *Aeromonas hydrophila* through Multiple Modulation of Physical and Immune Protective Capacity

**DOI:** 10.3390/ijms241411243

**Published:** 2023-07-08

**Authors:** Yao Zhang, Xiao-Qiu Zhou, Wei-Dan Jiang, Pei Wu, Yang Liu, Hong-Mei Ren, Xiao-Wan Jin, Lin Feng

**Affiliations:** 1 Animal Nutrition Institute, Sichuan Agricultural University, Chengdu 611100, China; zhangyao@stu.sicau.edu.cn (Y.Z.);; 2 Fish Nutrition and Safety Production University Key Laboratory of Sichuan Province, Sichuan Agricultural University, Chengdu 611100, China; 3 Key Laboratory for Animal Disease-Resistant Nutrition of China Ministry of Education, Sichuan Agricultural University, Chengdu 611100, China

**Keywords:** skin, mucosal barrier, antioxidant capacity, apoptosis, tight junction, immunity, vitamin D

## Abstract

The vertebrate mucosal barrier comprises physical and immune elements, as well as bioactive molecules, that protect organisms from pathogens. Vitamin D is a vital nutrient for animals and is involved in immune responses against invading pathogens. However, the effect of vitamin D on the mucosal barrier system of fish, particularly in the skin, remains unclear. Here, we elucidated the effect of vitamin D supplementation (15.2, 364.3, 782.5, 1167.9, 1573.8, and 1980.1 IU/kg) on the mucosal barrier system in the skin of grass carp (*Ctenopharyngodon idella*) challenged with *Aeromonas hydrophila*. Dietary vitamin D supplementation (1) alleviated *A. hydrophila*-induced skin lesions and inhibited oxidative damage by reducing levels of reactive oxygen species, malondialdehyde, and protein carbonyl; (2) improved the activities and transcription levels of antioxidant-related parameters and nuclear factor erythroid 2-related factor 2 signaling; (3) attenuated cell apoptosis by decreasing the mRNA and protein levels of apoptosis factors involved death receptor and mitochondrial pathway processes related to p38 mitogen-activated protein kinase and c-Jun N-terminal kinase signaling; (4) improved tight junction protein expression by inhibiting myosin light-chain kinase signaling; and (5) enhanced immune barrier function by promoting antibacterial compound and immunoglobulin production, downregulating pro-inflammatory cytokine expression, and upregulating anti-inflammatory cytokines expression, which was correlated with nuclear factor kappa B and the target of rapamycin signaling pathways. Vitamin D intervention for mucosal barrier via multiple signaling correlated with vitamin D receptor a. Overall, these results indicate that vitamin D supplementation enhanced the skin mucosal barrier system against pathogen infection, improving the physical and immune barriers in fish. This finding highlights the viability of vitamin D in supporting sustainable aquaculture.

## 1. Introduction

Recently, the risk of disease outbreaks in farmed fish species has become high due to the intensification and industrialization of the aquaculture industry, posing a considerable challenge to sustainable aquaculture [1]. Fish are continuously exposed to pathogens in the aquatic environment and have developed organs and barrier systems for survival, one of which is the skin. Fish skin represents a typical mucosal system that separates the internal and external environments and serves as both a physical and immune barrier to protect the organism against pathogens. However, the skin is also an ideal entry port for several opportunistic pathogens, and resulting infections can cause substantial economic losses for the aquaculture industry [2]. Therefore, promoting skin mucosal barrier function is important for sustainable disease-resistant in aquaculture.

Skin is the largest organ in the body, covers the entire body surface, and is vital for communication with the external environment. In particular, fish skin is a multipurpose tissue that plays salient roles in maintaining body shape, protecting against damage and infection, and ensuring osmotic balance [3]. Nutrition is thought to play a role in the response of mucosal systems to pathogens. Our previous work demonstrated that vitamin A modulated mucosal barrier function in fish gills [4]. Vitamin D, an essential nutrient for animals, is supplied via dietary intake and accumulates in fish throughout their lifetimes [5]. Several recent studies of aquatic animals have shown that vitamin D affects inflammatory responses in the intestines [6], lipid metabolism in the liver [7], glucose homeostasis in the hepatopancreas [8], and immunity in the head kidney [9]. However, no study has focused on whether vitamin D protects the mucosal system of functional organs during pathogenic infection. Dietary vitamin D intake has been shown to promote fish growth [10]. Fish growth is closely related to disease resistance, which in turn is associated with the physical and immune barrier functions of the skin [11]. Therefore, exploring the relationship between vitamin D and the skin mucosal system is necessary to promote and advance nutritional regulation in aquaculture.

The mucosal system consists of physical and immune barriers, and the physical barriers are controlled by processes such as antioxidation, cell apoptosis, and tight junction regulation [12]. Vitamin D was shown to alleviate oxidative stress, apoptosis, tight junction damage, and intestinal inflammation in yellow catfish (*Pelteobagrus fulvidraco*) after lipopolysaccharide challenge [13]. Additionally, vitamin D improved the antioxidant capacity in crabs (*Eriocheir sinensis*) by enhancing antioxidant enzyme expression in the hepatopancreas and intestine [14]. However, gaps in the current literature remain regarding the role of vitamin D in cell apoptosis and tight junction regulation, along with the molecular mechanisms by which vitamin D acts under pathogen infection. A previous study showed that apoptosis promoters and effectors are enhanced by p38 mitogen-activated protein kinase (MAPK) and c-Jun N-terminal kinase (JNK) [15]. Tight junctions are crucial for barrier function in various tissues (e.g., skin) and are composed of transmembrane proteins, including occludins and claudins [16], which are regulated by myosin light-chain kinase (MLCK) signaling [17]. Despite these findings, the specific mechanism by which vitamin D affects cell apoptosis and tight junctions should be investigated.

Immune barriers are crucial for structural integrity in fish and involve antimicrobial compounds and inflammatory cytokines [18]. The regulation of immune function continues to be the most well-recognized action of vitamin D. Intracrine synthesis of vitamin D by macrophages and dendritic cells stimulates the expression of antimicrobial compounds and plays a pivotal role in mediating T-cell responses, leading to the suppression of inflammatory cells and concomitant induction of immune responses [19]. Our previous study demonstrated that vitamin D enhanced the immune response in the head, kidney, and spleen in grass carp (*Ctenopharyngodon idella*) [9]. Unlike these organs, however, fish skin contains mucosa-associated lymphoid tissue, and its goblet and club cells secrete mucus containing a wide range of bioactive substances that act as humoral, innate immune factors and play important roles in inhibiting the entry of pathogens [20,21]. However, no studies have investigated the efficacy of vitamin D in regulating the immune response of fish skin.

Vitamin D-induced biochemical effects are mediated by its receptor (vitamin D receptor, VDR), which possesses two unique isoforms (VDRa and VDRb) in fish [22]. In VDR-deficient mice, high apoptotic cell counts were observed in the small intestine [23]. Moreover, transcription levels of claudin-2, claudin-4, and claudin-18 were reduced in the lungs of VDR-deficient mice [24]. In response to vitamin D, VDR induces the expression of antimicrobial peptides and inflammatory cytokines in rats [25]. Despite these findings in mammals, the interactions between vitamin D, VDR isoforms, and skin mucosal barriers in fish have not been extensively studied. Moreover, our unpublished data indicate that VDR is expressed in the skin of grass carp. However, the role of this VDR expression following pathogen infection is largely unknown in fish. Thus, elucidating the underlying molecular mechanisms is critical to understand the function of vitamin D during infection.

In view of this, we set out to examine whether vitamin D supplementation affects skin mucosal barrier function in fish. An economically important species in China, grass carp have been introduced in over 100 countries [26]. The global production of grass carp exceeded over 50 million tons in 2019 [27] and reached 5 million tons in China, accounting for 21.54% of the total freshwater aquaculture production in 2020 [28]. The intensive high-density aquaculture practices of recent years have made grass carp susceptible to various pathogens. Among these pathogens, *Aeromonas hydrophila* causes the most frequently occurring diseases in grass carp aquaculture [9]. *A. hydrophila* infection has led to inflammatory cell infiltration and microvillus effacement in the intestine [29,30], lymphocyte necrosis, and blood vessel necrosis in head kidney and spleen [9], and hemorrhage in the skin of grass carp [31]. To this end, for the first time, we examined the roles and potential signaling mechanisms of vitamin D and VDR in antioxidant systems, cell apoptosis, tight junction proteins, antibacterial compounds, and inflammatory cytokines in fish skin challenged by *A. hydrophila* infection. We aimed to identify key vitamin D-related molecular pathways related to skin mucosa regulation. Our findings provide a rationale for nutrient intervention practices that will promote sustainable aquaculture.

## 2. Results

### 2.1. Disease-Resistant Phenotypes

**Figure 1:**
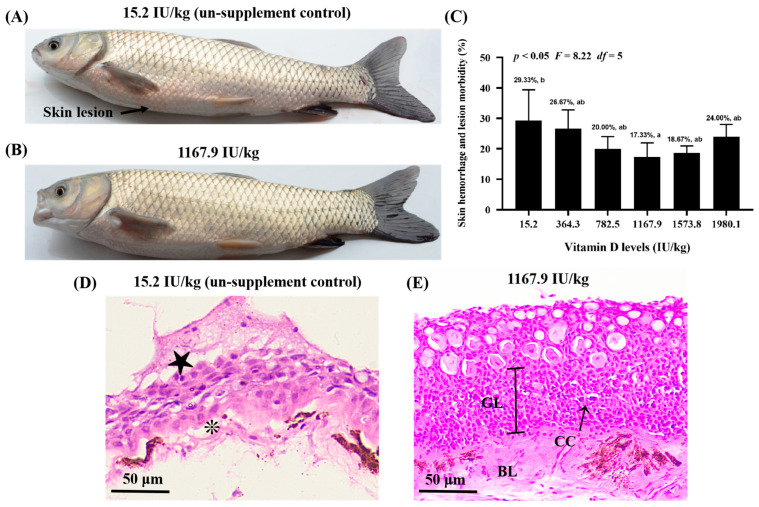
Vitamin D alleviates skin lesion morbidity in grass carp (*Ctenopharyngodon idella*) after infection with *Aeromonas hydrophila*. Differences in the severity of skin lesions in grass carp fed diets with 15.2 (**A**) and 1167.9 IU/kg (**B**) vitamin D supplementation. Arrowhead indicates the skin lesion. (**C**) Effects of available vitamin D levels on skin lesion severity in grass carp after infection with *A. hydrophila*. Hematoxylin and eosin staining of fish skin in the 15.2 (**D**) and 1167.9 IU/kg (**E**) vitamin D supplementation group. Epithelial ulceration (★), disintegration of tissue structure (❊), germinal layer (GL), basal layer (GL), club cell (CC). Values are the means ± SD, represented by vertical bars. Values with different letters are significantly different (*p* < 0.05).

Skin lesions were observed in the control group (VD15.2); however, *A. hydrophila*-induced skin lesions were attenuated in the VD1167.9 group (Figure 1A,B). Additionally, vitamin D supplementation reduced skin lesion-induced morbidity compared with that in the control group (29.33%), with the lowest morbidity (17.33%) observed in the VD11679 group (Figure 1C). The histopathological results (Figure 1D,E) revealed that vitamin D deficiency was associated with epithelial ulceration and disintegration of tissue structure in the skin of grass carp, and vitamin D supplementation alleviated the pathology.

### 2.2. Oxidative Damage Biomarkers and Antioxidant Enzymes

**Figure 2:**
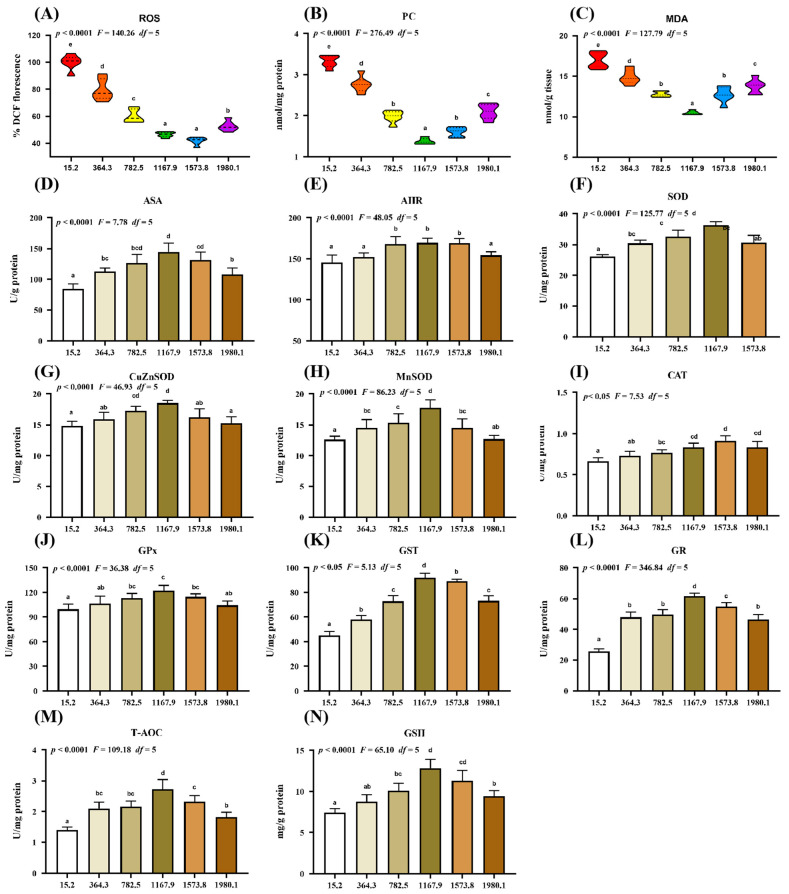
Impact of dietary vitamin D on barrier function in the skin of grass carp (*Ctenopharyngodon idella*) after infection with *A. hydrophila*. (**A**–**C**) Biomarkers of oxidative damage: ROS, reactive oxygen species (% DCF fluorescence); MDA, malondialdehyde (nmol/g tissue); PC, protein carbonyl (nmol/mg protein). (**D**–**N**) Antioxidant-related parameters: ASA, anti-superoxide anion (U/g protein); AHR, anti-hydroxy radical (U/mg protein); SOD, superoxide dismutase (U/mg protein); CuZnSOD, copper/zinc superoxide dismutase (U/mg protein); MnSOD, manganese superoxide dismutase (U/mg protein); CAT, catalase (U/mg protein); GPx, glutathione peroxidase (U/mg protein); GST, glutathione S-transferase (U/mg protein); GR, glutathione reductase (U/mg protein); T-AOC, Total antioxidant capacity (U/mg protein); GSH, glutathione (mg/g protein). N = 6 for each vitamin D level. Different letters above bars indicate significant differences (*p* < 0.05).

The effect of vitamin D supplementation on oxidative damage and the antioxidative system was examined (Figure 2). Compared with the control group, vitamin D supplementation caused a dose-dependent decrease in the levels of oxidative damage biomarkers, including malondialdehyde (MDA), protein carbonyl (PC), and reactive oxygen species (ROS), in the fish (Figure 2A–C). Specifically, the lowest ROS (*p*_quadratic_ < 0.0001, *F*_quadratic_ = 140.26, *p*_Linear_ < 0.0001, *F*_Linear_ = 473.72, *df* = 5), PC (*p*_quadratic_ < 0.0001, *F*_quadratic_ = 276.49, *p*_Linear_ < 0.0001, *F*_Linear_ = 375.61, *df* = 5), and MDA (*p*_quadratic_ < 0.0001, *F*_quadratic_ = 127.79, *p*_Linear_ < 0.0001, *F*_Linear_ = 88.54, *df* = 5) levels were observed in the VD1573.8, VD1167.9, and VD1167.9 groups, respectively. Additionally, vitamin D supplementation caused a significant increase in the levels of the anti-superoxide anion (ASA; *p*_quadratic_ < 0.0001, *F* = 7.78, *df* = 5) and anti-hydroxy radical (AHR; *p*_quadratic_ < 0.0001, *F*_quadratic_ = 48.05, *df* = 5), both of which peaked in the VD1167.9 group.

The activities of antioxidant enzymes reflect the antioxidant capacity and free radical scavenging activity in the fish. Compared with those in control group, vitamin D supplementation increased the activities of superoxide dismutase (SOD; *p*_quadratic_ < 0.0001, *F*_quadratic_ = 125.77, *p*_Linear_ < 0.05, *F*_Linear_ = 6.37, *df* = 5), CuZnSOD (*p*_quadratic_ < 0.0001, *F*_quadratic_ = 46.93, *p*_Linear_ = 0.21, *F*_Linear_ = 1.62, *df* = 5), MnSOD (*p*_quadratic_ < 0.0001, *F*_quadratic_ = 86.23, *p*_Linear_ < 0.05, *F*_Linear_ = 5.33, *df* = 5), glutathione peroxidase (GPx; *p*_quadratic_ < 0.0001, *F*_quadratic_ = 36.38, *p*_Linear_ < 0.05, *F*_Linear_ = 7.20, *df* = 5), glutathione S-transferase (GST; *p*_quadratic_ < 0.05, *F*_quadratic_ = 5.13, *p*_Linear_ < 0.01, *F*_Linear_ = 12.73, *df* = 5), glutathione reductase (GR; *p*_quadratic_ < 0.0001, *F*_quadratic_ = 346.84, *p*_Linear_ < 0.001, *F*_Linear_ = 219.03, *df* = 5), total antioxidant capacity (T-AOC; *p*_quadratic_ < 0.0001, *F*_quadratic_ = 109.18, *p*_Linear_ < 0.01, *F*_Linear_ = 22.97, *df* = 5), and glutathione (GSH; *p*_quadratic_ < 0.0001, *F*_quadratic_ = 65.10, *p*_Linear_ < 0.001, *F*_Linear_ = 42.82, *df* = 5), all of which peaked in the VD1167.9 group, as well as catalase (CAT) activity (*p*_quadratic_ < 0.05, *F*_quadratic_ = 7.53, *p*_Linear_ < 0.001, *F*_Linear_ = 64.98, *df* = 5), which peaked in the VD1573.8 group.

### 2.3. Key Regulatory Genes Involved in the Physical Barrier Function of Skin

#### 2.3.1. Key Regulatory Genes for Antioxidant-Related Parameters

**Figure 3:**
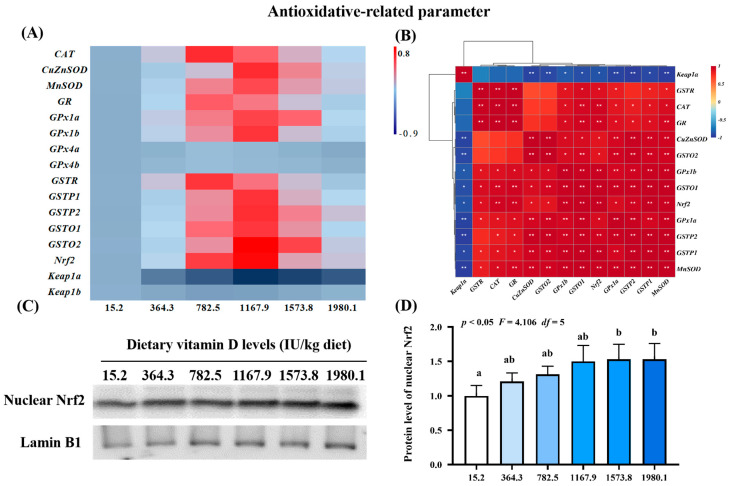
Heat map and correlation analysis of antioxidative−related parameters (**A**,**B**) and Nrf2 protein levels (**C**,**D**) in the skin of on−growing grass carp (*Ctenopharyngodon idella*) administered vitamin D after infection with *A. hydrophila*. Nrf2, nuclear factor E2−related factor 2; Keap1, kelch−like ECH−associated protein 1. N = 6 for gene expression and N = 3 for Western blotting analysis. Different letters above bars indicate significant differences (*p* < 0.05); red indicates upregulation; blue indicates downregulation; * presents *p* < 0.05, ** presents *p* < 0.01.

**Figure 4:**
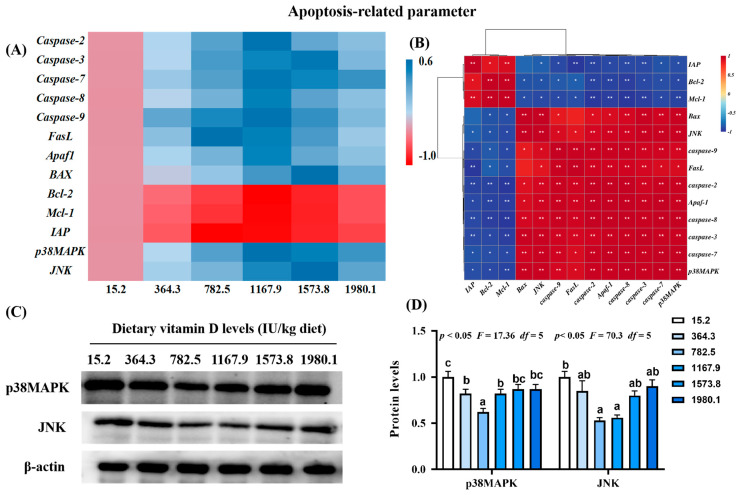
Heat map and correlation analysis of apoptosis−related parameters (**A**,**B**) and protein levels of p38MAPK and JNK (**C**,**D**) in the skin of on−growing grass carp (*Ctenopharyngodon idella*) administered vitamin D after infection with *A. hydrophila*. Caspase, cysteinyl aspartate specific proteinase; FasL, Fas ligand; APaf1, apoptotic protease activating factor−1; Bcl−2, B−cell lymphoma−2; BAX, Bcl-2 associated X; Mcl−1, myeloid cell leukemia−1; IAP, inhibitor of apoptosis protein; p38MAPK, mitogen−activated protein kinases; JNK, c-Jun N−terminal kinase; N = 6 for gene expression and N = 3 for Western blotting analysis; different letters above bars indicate significant differences (*p* < 0.05); red indicates upregulation; blue indicates downregulation; * presents *p* < 0.05, ** presents *p* < 0.01.

**Figure 5:**
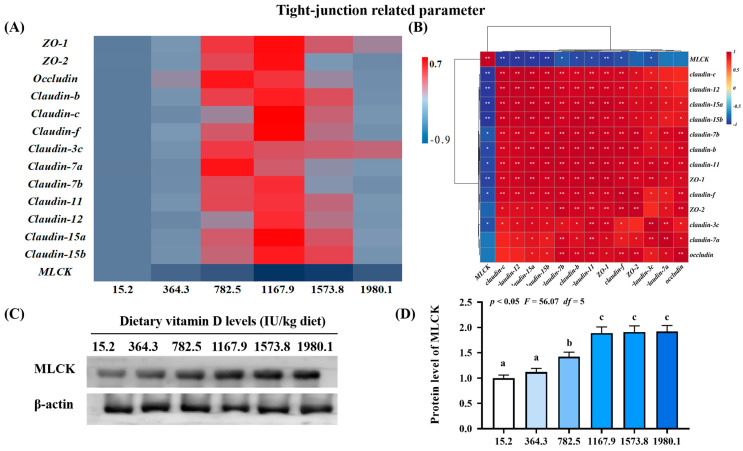
Heat map and correlation analysis of apoptosis−related parameters (**A**,**B**) and protein levels of MLCK (**C**,**D**) in the skin of on−growing grass carp (*Ctenopharyngodon idella*) administered vitamin D after infection with *A. hydrophila*. ZO−1, zonula occludens; MLCK, myosin light chain Kinase; N = 6 for gene expression and N = 3 for western blotting analysis; different letters above bars indicate significant differences (*p* < 0.05); red indicates upregulation; blue indicates downregulation; * presents *p* < 0.05, ** presents *p* < 0.01.

To further confirm the effect of vitamin D on the physical barrier function of skin during *A. hydrophila* infection, we examined the expression of antioxidant-, apoptosis-, and tight junction-related genes and protein levels of key molecules (Figure 3, Figure 4 and Figure 5). Compared with levels in the control group (VD15.2), vitamin D supplementation significantly increased the expression of antioxidant-related genes (Figure 3A), including *MnSOD* (*p*_quadratic_ < 0.05, *F*_quadratic_ = 12.29, *p*_Linear_ < 0.05, *F*_Linear_ = 6.32, *df* = 5), *CuZnSOD* (*p*_quadratic_ < 0.05, *F*_quadratic_ = 12.19, *p*_Linear_ < 0.01, *F*_Linear_ = 10.89, *df* = 5), *GPx1a* (*p*_quadratic_ < 0.001, *F*_quadratic_ = 16.27, *p*_Linear_ < 0.05, *F*_Linear_ = 6.39, *df* = 5), *GPx1b* (*p*_quadratic_ < 0.001, *F*_quadratic_ = 17.77, *p*_Linear_ = 0.10, *F*_Linear_ = 3.03, *df* = 5), *GSTP1* (*p*_quadratic_ < 0.001, *F*_quadratic_ = 13.59, *p*_Linear_ < 0.05, *F*_Linear_ = 5.28, *df* = 5), *GSTP2* (*p*_quadratic_ < 0.001, *F*_quadratic_ = 15.10, *p*_Linear_ < 0.01, *F*_Linear_ = 9.85, *df* = 5), *GSTo1* (*p*_quadratic_ < 0.001, *F*_quadratic_ = 18.68, *p*_Linear_ < 0.05, *F*_Linear_ = 4.5, *df* = 5), *GSTo2* (*p*_quadratic_ < 0.001, *F*_quadratic_ = 25.81, *p*_Linear_ < 0.01, *F*_Linear_ = 9.08, *df* = 5), and nuclear factor erythroid 2-related factor 2 (*Nrf2*; *p*_quadratic_ < 0.001, *F*_quadratic_ = 18.95, *p*_Linear_ < 0.05, *F*_Linear_ = 4.65, *df* = 5), all of which peaked in the VD1167.9 group and decreased at higher vitamin D concentrations (1167.9–1980.1 IU/kg). Additionally, *CAT* (*p*_quadratic_ < 0.001, *F*_quadratic_ = 13.86, *p*_Linear_ = 0.18, *F*_Linear_ = 1.90, *df* = 5), *GSTR* (*p*_quadratic_ < 0.001, *F*_quadratic_ = 21.67, *p*_Linear_ = 0.49, *F*_Linear_ = 0.47, *df* = 5), and *GR* (*p*_quadratic_ < 0.001, *F*_quadratic_ = 16.27, *p*_Linear_ = 0.19, *F*_Linear_ = 1.72, *df* = 5) expression levels were significantly increased by vitamin D supplement and peaked in the VD782.5 group, whereas *Keap1a* (*p*_quadratic_ < 0.001, *F*_quadratic_ = 22.89, *p*_Linear_ < 0.0001, *F*_Linear_ = 32.74, *df* = 5) expression showed a decreasing trend, with the lowest expression observed in the VD1167.9 group. In contrast, *GPx4a* (*p*_quadratic_ = 0.26, *F*_quadratic_ = 1.34, *p*_Linear_ = 0.81, *F*_Linear_ = 0.06, *df* = 5)*, -4b* (*p*_quadratic_ = 0.31, *F*_quadratic_ = 1.04, *p*_Linear_ = 0.90, *F*_Linear_ = 0.02, *df* = 5), and *Keap1b* (*p*_quadratic_ = 0.21, *F*_quadratic_ = 1.64, *p*_Linear_ = 0.89, *F*_Linear_ = 0.02, *df* = 5) expression levels were not significantly affected (*p* > 0.05) by vitamin D supplementation. Correlation analysis showed that the mRNA levels of most antioxidant enzymes were positively correlated with *Nrf2* and negatively correlated with *Keap1a* levels (Figure 3B). Nrf2 protein expression (*p*_quadratic_ < 0.05, *F*_quadratic_ = 4.106, *p*_Linear_ = 0.09, *F*_Linear_ = 5.66, *df* = 5) increased with vitamin D supplementation, peaking in the VD1573.8 group (Figure 3C,D).

#### 2.3.2. Key Regulatory Gene for Apoptosis-Related Parameters

There were decreases in the expression of pro-apoptosis genes, including cysteinyl aspartate specific proteinase (*Caspase*)*-2* (*p*_quadratic_ < 0.001, *F*_quadratic_ = 34.74, *p*_Linear_ < 0.0001, *F*_Linear_ = 26.03, *df* = 5), *-7* (*p*_quadratic_ < 0.001, *F*_quadratic_ = 35.48, *p*_Linear_ < 0.0001, *F*_Linear_ = 66.84, *df* = 5), *-8* (*p*_quadratic_ < 0.001, *F*_quadratic_ = 23.07, *p*_Linear_ < 0.0001, *F*_Linear_ = 23.25, *df* = 5), *-9* (*p*_quadratic_ < 0.001, *F*_quadratic_ = 43.23, *p*_Linear_ < 0.0001, *F*_Linear_ = 21.08, *df* = 5), and apoptotic protease activating factor-1 (*Afaf1*; *p*_quadratic_ < 0.001, *F*_quadratic_ = 29.06, *p*_Linear_ < 0.0001, *F*_Linear_ = 26.16, *df* = 5) in the VD1167.9 group compared with the control group (Figure 4A). The VD1573.8 group was associated with significantly downregulated mRNA levels of *Caspase-3* (*p*_quadratic_ < 0.001, *F*_quadratic_ = 39.15, *p*_Linear_ < 0.0001, *F*_Linear_ = 44.99, *df* = 5), *BAX* (*p*_quadratic_ < 0.001, *F*_quadratic_ = 14.45, *p*_Linear_ < 0.0001, *F*_Linear_ = 59.38, *df* = 5), and c-Jun N-terminal kinase (*JNK*; *p*_quadratic_ < 0.001, *F*_quadratic_ = 22.29, *p*_Linear_ < 0.0001, *F*_Linear_ = 46.52, *df* = 5) relative to values in the VD15.2 group. The lowest expression level of Fas ligand (*FasL*) was found in the VD782.5 group. In contrast, the expression of B-cell lymphoma-2 (*Bcl-2*; *p*_quadratic_ < 0.05, *F*_quadratic_ = 11.63, *p*_Linear_ < 0.01, *F*_Linear_ = 9.08, *df* = 5) and myeloid cell leukemia-1 (*Mcl-1; p*_quadratic_ < 0.001, *F*_quadratic_ = 14.39, *p*_Linear_ < 0.001, *F*_Linear_ = 7.90, *df* = 5) increased with increasing concentration of vitamin D, peaking in the VD1167.9 group, whereas inhibitor of apoptosis (*IAP*; *p*_quadratic_ < 0.0001, *F*_quadratic_ = 14.39, *p*_Linear_ < 0.05, *F*_Linear_ = 6.23, *df* = 5) expression peaked in the VD782.5 group. The expression of p38*MAPK* (*p*_quadratic_ < 0.001, *F*_quadratic_ = 27.42, *p*_Linear_ < 0.0001, *F*_Linear_ = 56.26, *df* = 5) decreased with increasing vitamin D concentration. p38MAPK and JNK mRNA expression levels were positively correlated with pro-apoptotic gene expression and negatively correlated with anti-apoptotic factors (Figure 4B). Compared with that in the control group, the protein expression of p38MAPK (*p*_quadratic_ < 0.05, *F*_quadratic_ = 17.36, *p*_Linear_ = 0.29, *F*_Linear_ = 1.64, *df* = 5) and JNK (*p*_quadratic_ < 0.05, *F*_quadratic_ = 70.3, *p*_Linear_ = 0.13, *F*_Linear_ = 4.07, *df* = 5) decreased with enhanced vitamin D supplementation, and the lowest values were found in VD782.5 group (Figure 4C,D).

#### 2.3.3. Key Regulatory Gene for Tight Junction-Related Parameters

The VD1167.9 group elevated expression levels of zonula occludens (*ZO*)*-1* (*p*_quadratic_ < 0.05, *F*_quadratic_ = 8.19, *p*_Linear_ < 0.05, *F*_Linear_ = 6.30, *df* = 5) and *-2* (*p*_quadratic_ < 0.01, *F*_quadratic_ = 15.02, *p*_Linear_ = 0.45, *F*_Linear_ = 0.59, *df* = 5) and *claudin-b* (*p*_quadratic_ < 0.001, *F*_quadratic_ = 12.34, *p*_Linear_ < 0.05, *F*_Linear_ = 7.22, *df* = 5), *-c* (*p*_quadratic_ < 0.01, *F*_quadratic_ = 14.71, *p*_Linear_ = 0.06, *F*_Linear_ = 3.96, *df* = 5), *-f* (*p*_quadratic_ < 0.0001, *F*_quadratic_ = 25.39, *p*_Linear_ =0.09, *F*_Linear_ = 3.33, *df* = 5), *-7b* (*p*_quadratic_ < 0.01, *F*_quadratic_ = 9.68, *p*_Linear_ = 0.19, *F*_Linear_ = 1.86, *df* = 5), *-11* (*p*_quadratic_ < 0.05, *F*_quadratic_ = 5.36, *p*_Linear_ < 0.05, *F*_Linear_ = 7.72, *df* = 5), *-12* (*p*_quadratic_ < 0.05, *F*_quadratic_ = 4.96, *p*_Linear_ < 0.05, *F*_Linear_ = 7.62, *df* = 5), *-15a* (*p*_quadratic_ < 0.01, *F*_quadratic_ = 9.52, *p*_Linear_ < 0.05, *F*_Linear_ = 6.76, *df* = 5), and -15b (*p*_quadratic_ =0.07, *F*_quadratic_ = 3.74, *p*_Linear_ = 0.45, *F*_Linear_ = 0.6, *df* = 5) (Figure 5A). Similarly, vitamin D supplementation increased *occludin* (*p*_quadratic_ < 0.05, *F*_quadratic_ = 6.46, *p*_Linear_ = 0.60, *F*_Linear_ = 0.28, *df* = 5) and *claudin*-*3c* (*p*_quadratic_ < 0.05, *F*_quadratic_ = 8.55, *p*_Linear_ = 0.54, *F*_Linear_ = 0.39, *df* = 5) and -7a (*p*_quadratic_ < 0.05, *F*_quadratic_ = 4.45, *p*_Linear_ = 0.05, *F*_Linear_ = 4.70, *df* = 5) expression, which peaked in the VD782.5 group. In contrast, vitamin D supplementation decreased the expression of the key regulator of tight junctions *MLCK* (*p*_quadratic_ < 0.001, *F*_quadratic_ = 23.13, *p*_Linear_ < 0.001, *F*_Linear_ = 24.88, *df* = 5), with the lowest level observed in the VD1167.9 group. Correlation analysis showed that most tight junction genes were negatively correlated with *MLCK* levels (Figure 5B). MLCK protein level (*p*_quadratic_ < 0.05, *F*_quadratic_ = 56.07, *p*_Linear_ < 0.01, *F*_Linear_ = 92.92, *df* = 5) increased with vitamin D supplementation, peaking in the VD1573.8 group (Figure 5C,D).

### 2.4. Immune-Related Parameters and Key Regulatory Genes of Skin Immune Barrier Function

**Figure 6:**
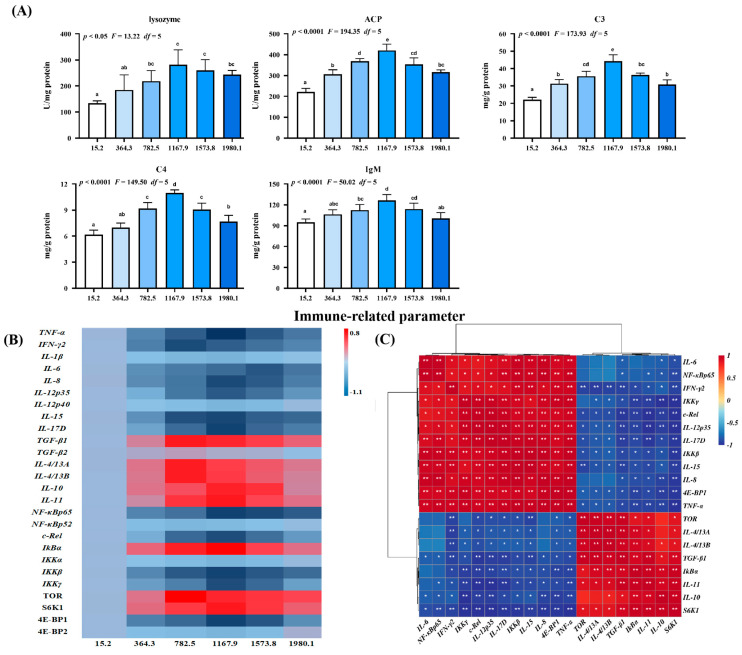
Immune-related parameters (**A**), heat map (**B**), and correlation analysis (**C**) in the skin of grass carp (*Ctenopharyngodon idella*) administrated with vitamin D after *A. hydrophila* infection. ACP, acid phosphatase (U/mg protein); C3, complement 3 (mg/g protein); C4, complement 4 (mg/g protein); IgM, immunoglobulin M (mg/g protein); TNF, tumor necrosis factor; IFN, interferon; IL, interleukin; TGF, transforming growth factor; NF−κB, nuclear factor kappa B; IκBα, inhibitor of NF−κB; IKK, inhibitor of kappa B kinase; TOR, target of rapamycin; S6K1, ribosomal protein S6 kinase 1; 4E−BP, 4E−binding protein 1. N = 6 for biochemical analysis and gene expression; N = 3 for western blotting analysis; different letters above bars indicate significant differences (*p* < 0.05); red indicates upregulation; blue indicates downregulation; * presents *p* < 0.05, ** presents *p* < 0.01.

The effect of vitamin D on immune barrier function in fish skin during *A. hydrophila* infection was examined (Figure 6). Vitamin D supplementation increased the activities of lysosomes (*p*_quadratic_ < 0.05, *F*_quadratic_ = 13.22, *p*_Linear_ < 0.0001, *F*_Linear_ = 35.31, *df* = 5), acid phosphatase (ACP; *p*_quadratic_ < 0.0001, *F*_quadratic_ = 194.35, *p*_Linear_ < 0.0001, *F*_Linear_ = 80.96, *df* = 5), complement 3 (C3; *p*_quadratic_ < 0.0001, *F*_quadratic_ = 173.93, *p*_Linear_ < 0.0001, *F*_Linear_ = 64.63, *df* = 5), C4 (*p*_quadratic_ < 0.0001, *F*_quadratic_ = 149.50, *p*_Linear_ < 0.0001, *F*_Linear_ = 57.85, *df* = 5), and immunoglobulin M (IgM; *p*_quadratic_ < 0.0001, *F*_quadratic_ = 50.02, *p*_Linear_ < 0.05, *F*_Linear_ = 6.3, *df* = 5) compared with those in the control group, peaking in the VD1167.9 group (Figure 6A).

Additionally, we examined the effect of vitamin D on skin immune barrier function in *A. hydrophila*−challenged fish and the expression of inflammatory cytokines and related signaling genes (Figure 6B). Vitamin D significantly downregulated the expression of interleukin (*IL*)*-8* (*p*_quadratic_ < 0.001, *F*_quadratic_ = 25.22, *p*_Linear_ < 0.001, *F*_Linear_ = 26.36, *df* = 5), *-15* (*p*_quadratic_ < 0.0001, *F*_quadratic_ = 30.85, *p*_Linear_ < 0.01, *F*_Linear_ = 38.08, *df* = 5), -12p35 (*p*_quadratic_ = 0.26, *F*_quadratic_ = 1.31, *p*_Linear_ = 0.71, *F*_Linear_ = 0.13, *df* = 5), *-17D* (*p*_quadratic_ < 0.0001, *F*_quadratic_ = 25.34, *p*_Linear_ < 0.001, *F*_Linear_ = 28.48, *df* = 5) and tumor necrosis factor (*TNF*)*-α* (*p*_quadratic_ < 0.0001, *F*_quadratic_ = 41.36, *p*_Linear_ < 0.0001, *F*_Linear_ = 32.31, *df* = 5) compared with the control group, with the lowest expression levels observed in the VD1167.9 group. Similarly, vitamin D supplementation downregulated interferon (*IFN*)*-γ2* (*p*_quadratic_ < 0.001, *F*_quadratic_ = 43.67, *p*_Linear_ < 0.001, *F*_Linear_ = 17.18, *df* = 5) and *IL-6* (*p*_quadratic_ < 0.001, *F*_quadratic_ = 17.09, *p*_Linear_ < 0.001, *F*_Linear_ = 35.37, *df* = 5) expression, with the lowest expression levels observed in the VD782.5 and VD1573.8 groups, respectively; however, *IL-1β* (*p*_quadratic_ = 0.14, *F*_quadratic_ = 2.23, *p*_Linear_ = 0.70, *F*_Linear_ = 0.15, *df* = 5) and *-12p40* (*p*_quadratic_ < 0.001, *F*_quadratic_ = 25.11, *p*_Linear_ < 0.001, *F*_Linear_ = 20.62, *df* = 5) expression levels were not affected by vitamin D supplementation. In contrast, vitamin D supplementation increased the expression of anti-inflammatory cytokines, including *TGF-β1* (*p*_quadratic_ < 0.0001, *F*_quadratic_ = 15.32, *p*_Linear_ < 0.05, *F*_Linear_ = 7.22, *df* = 5), *IL-14/13A* (*p*_quadratic_ < 0.01, *F*_quadratic_ = 11.81, *p*_Linear_ = 0.19, *F*_Linear_ = 1.78, *df* = 5), and *-14/13B* (*p*_quadratic_ < 0.001, *F*_quadratic_ = 18.66, *p*_Linear_ = 0.18, *F*_Linear_ = 1.83, *df* = 5), peaking in the VD782.5 group. Similarly, *IL-10* (*p*_quadratic_ < 0.001, *F*_quadratic_ = 25.35, *p*_Linear_ < 0.01, *F*_Linear_ = 7.86, *df* = 5) and -*11* (*p*_quadratic_ < 0.001, *F*_quadratic_ = 23.84, *p*_Linear_ < 0.01, *F* _Linear_ = 9.95, *df* = 5) expression levels increased with increasing dietary vitamin D concentration, peaking in the VD1167.9 group; however, transforming growth factor (*TGF*)*-β2* (*p*_quadratic_ = 0.39, *F*_quadratic_ = 0.76, *p*_Linear_ = 0.75, *F*_Linear_ = 0.10, *df* = 5) expression was not affected by dietary vitamin D supplementation.

In the key regulatory signaling pathways, vitamin D supplementation downregulated the expression of nuclear factor kappa B (*NF-κB*) *p65* (*p*_quadratic_ < 0.0001, *F*_quadratic_ = 27.30, *p*_Linear_ < 0.0001, *F*_Linear_ = 59.13, *df* = 5), *C-Rel* (*p*_quadratic_ < 0.0001, *F*_quadratic_ = 40.28, *p*_Linear_ < 0.0001, *F*_Linear_ = 22.00, *df* = 5), an inhibitor of kappa B kinase *β (IKKβ)* (*p*_quadratic_ < 0.05, *F*_quadratic_ = 13.46, *p*_Linear_ < 0.05, *F*_Linear_ = 12.12, *df* = 5), and *IKKγ* (*p*_quadratic_ < 0.01, *F*_quadratic_ = 23.12, *p*_Linear_ < 0.05, *F*_Linear_ = 7.15, *df* = 5)*,* all of which were lowest in the VD1167.9 group. In contrast, the inhibitor of KBα (*IKBα*) (*p*_quadratic_ < 0.0001, *F*_quadratic_ = 31.75, *p*_Linear_ < 0.05, *F*_Linear_ = 5.89, *df* = 5) expression increased with increasing vitamin D concentration, peaking in the VD1167.9 group; however, *NF-κBp52* (*p*_quadratic =_ 0.21, *F*_quadratic_ = 1.67, *p*_Linear_ = 0.97, *F*_Linear_ = 0.01, *df* = 5) and *IKKα* (*p*_quadratic_ = 0.9252, *F*_quadratic_ = 0.01, *p*_Linear_ = 0.92, *F*_Linear_ = 0.01, *df* = 5) expressions were unaffected by vitamin D supplementation. Similarly, vitamin D supplementation increased target of rapamycin (*TOR; p*_quadratic_ < 0.05, *F*_quadratic_ = 3.19, *p*_Linear_ = 0.25, *F*_Linear_ = 1.6, *df* = 5) and ribosomal protein S6 kinase 1 (*S6K1*; *p*_quadratic_ < 0.01, *F*_quadratic_ = 14.66, *p*_Linear_ = 0.27, *F*_Linear_ = 1.46, *df* = 5) expression, which peaked in the VD782.5 and VD1167.9 groups, respectively, but downregulated 4E-binding protein 1 (*4E-BP1*; *p*_quadratic_ < 0.05, *F*_quadratic_ = 4.21, *p*_Linear_ = 0.16, *F*_Linear_ = 2.46, *df* = 5) expression, with the lowest level observed in the VD1167.9 group. However, *4E-BP2* (*p*_quadratic_ < 0.05, *F*_quadratic_ =13.75, *p*_Linear_ < 0.05, *F*_Linear_ = 7.57, *df* = 5) expression was not significantly affected by vitamin D supplementation. Correlation analysis showed that the expression of anti-inflammatory genes was positively correlated with *TOR* and *S6K1* expression but negatively correlated with *NF-κBp65* (Figure 6C).

**Figure 7:**
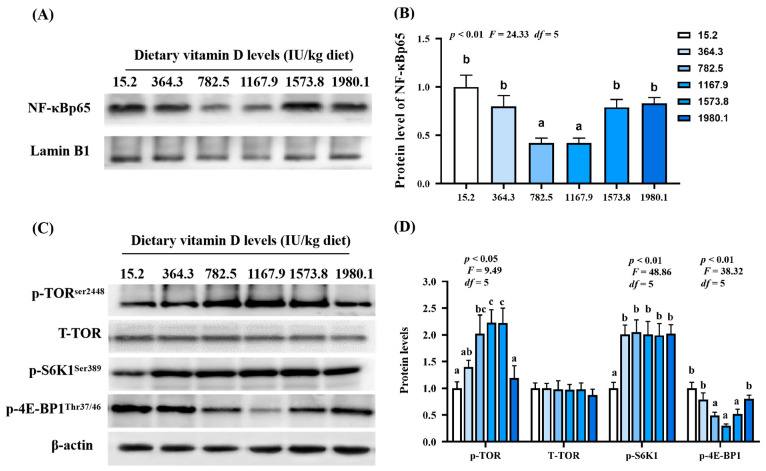
Protein levels of immune-related parameters in the skin of grass carp (*Ctenopharyngodon idella*) administrated with vitamin D after *A. hydrophila* infection. Protein bands (**A**) and analysis result (**B**) of NF−κB; Protein levels (**C**) and analysis results (**D**) of p−TOR, T−TOR^Ser2448^, p−S6K1^Ser389^, and p−4E−BP1^Thr37/46^. N = 3 for Western blotting analysis; different letters above bars indicate significant differences (*p* < 0.05).

As shown in Figure 7A, NF-κBp65 protein expression (*p*_quadratic_ < 0.01, *F*_quadratic_ = 24.33, *p*_Linear_ = 0.29, *F*_Linear_ = 1.64, *df* = 5) decreased with increasing vitamin D supplementation, and the lowest expression observed in the VD1167.9 group. Vitamin D supplementation significantly increased p-TOR^Ser2448^ (*p*_quadratic_ = 0.13, *F*_quadratic_ =4.20, *df* = 5) and p-S6K1^Ser389^ protein expression (*p*_quadratic_ < 0.01, *F*_quadratic_ =48.86, *p*_Linear_ < 0.01, *F*_Linear_ = 74.54, *df* = 5) compared to levels in the control (VD15.2) group. However, T-TOR protein expression was not affected. Additionally, p-4E-BP1^Thr37/46^ protein expression (*p*_quadratic_ < 0.01, *F*_quadratic_ = 38.32, *p*_Linear_ = 0.09, *F*_Linear_ = 5.73, *df* = 5) significantly decreased with increasing vitamin D concentration, and the lowest value was found in the VD1167.9 group (Figure 7B).

### 2.5. Expression of VDR

**Figure 8:**
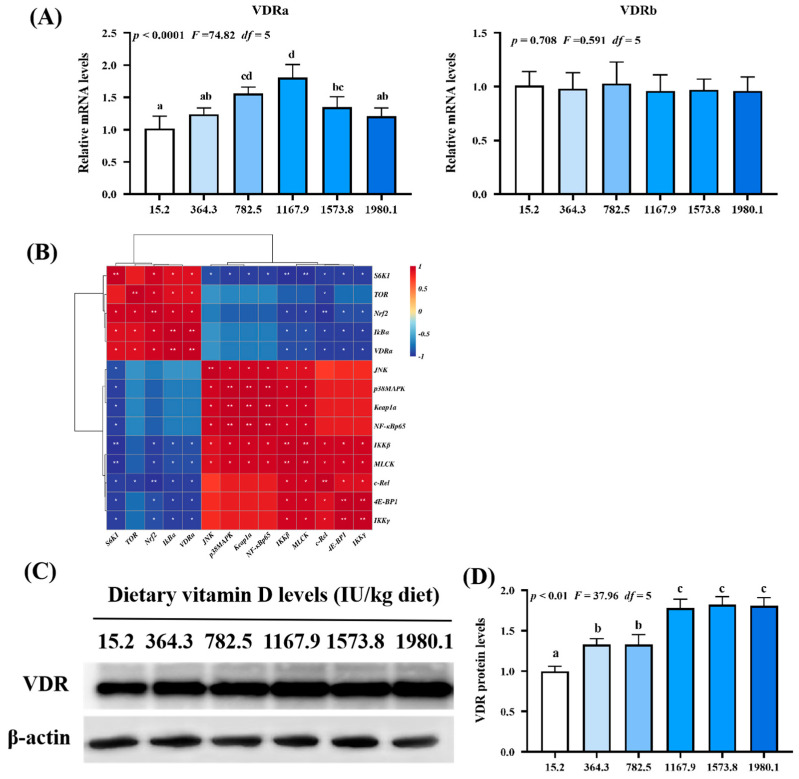
Relative mRNA levels (**A**), correlation analysis (**B**), protein levels (**C**) and analysis result (**D**) of VDR in the skin of on−growing grass carp (*Ctenopharyngodon idella*) administered vitamin D after infection with *A. hydrophila*. VDR, vitamin D receptor; N = 6 for gene expression and N = 3 for western blotting analysis; error bars indicate SD. Values with different letters are significantly different (*p* < 0.05); red indicates upregulation; blue indicates downregulation; * presents *p* < 0.05, ** presents *p* < 0.01.

As shown in Figure 8A, *VDRa* expression (*p*_quadratic_ < 0.0001, *F*_quadratic_ = 74.82, *p*_Linear_ < 0.01, *F*_Linear_ = 8.35, *df* = 5) was upregulated with increasing vitamin D supplementation, peaking in the VD1167.9 group, whereas *VDRb* expression (*p*_quadratic_ = 0.708, *F*_quadratic_ = 0.591, *p*_Linear_ = 0.49, *F*_Linear_ = 0.48, *df* = 5) was not significantly affected. Correlation analysis (Figure 8B) showed that *VDRa* expression was positively correlated with *Nrf2*, *TOR*, *S6K1*, *IκBα* expression but negatively correlated with *MLCK*, *4E-BP1*, *c-Rel*, *IKKβ*, and *IKKγ* expression. Compared with those in the un-supplemented control group, VDR protein level (*p*_quadratic_ < 0.01, *F*_quadratic_ = 37.96, *p*_Linear_ < 0.01, *F*_Linear_ = 71.35, *df* = 5) was significantly elevated under vitamin D supplementation (Figure 8C,D).

## 3. Discussion

### 3.1. Dietary Vitamin D Supplementation Enhanced Disease Resistance in Fish Skin

The health of fish skin is reflected in its ability to resist pathogens [11]. *A. hydrophila* is one of the most common pathogens in aquatic environments, which induces disturbance by destroying the mucosal barrier in fish skin [32]. In the present study, apparent skin lesions, epithelial ulceration and disintegration of tissue structure were induced by infection in the un-supplement control group. Vitamin D supplementation (1167.9 IU/kg) decreased skin lesion-morbidity compared with the control group (29.33%), with the lowest lesion-induced morbidity (17.33%) observed in the VD1167.9 group. This result indicated that dietary vitamin D prevented skin lesions, thus enhancing resistance against *A. hydrophila*. Generally, the health of the skin is tightly associated with physical and immune barriers. Therefore, we examined, for the first time, the influence of dietary vitamin D on physical and immune barriers in the skin of grass carp.

### 3.2. Dietary Vitamin D Supplementation Improved Physical Barrier Function in the Skin

#### 3.2.1. Dietary Vitamin D Supplementation Enhanced Antioxidant Capacity in the Skin

The physical barrier function of the skin is related to cellular and intercellular integrity and is reflected by antioxidant capacity, apoptosis, and tight junction. Under pathogenic infection, organisms produce and accumulate ROS, inducing DNA, lipid, and protein damage [33]. The degree of lipid peroxidation and protein oxidation can be determined using MDA and PC, respectively [34]. Organisms have developed complex antioxidant systems and use antioxidant enzymes to prevent disease- or stress-induced oxidative damage [35]. In the present study, dietary vitamin D decreased the levels of oxidative damage biomarkers (ROS, MDA, and PC) and enhanced the activity of antioxidant enzymes in infected fish. Under an acute lipopolysaccharide challenge, vitamin D pretreatment alleviated intestinal oxidative damage in yellow catfish [13]. Moreover, fish antioxidant systems can employ non-enzymatic (such as GSH) and enzymatic (like SOD, CAT, GPx, GST, and GR) antioxidants to protect functional organs from oxidative damage [36]. We observed that vitamin D improved antioxidant enzyme activity and corresponding gene expression in fish skin, which is consistent with findings in crabs [14]. These results indicate that the enhancement of vitamin D-mediated antioxidant capacity inhibits oxidative damage in fish skin. Additionally, vitamin D supplementation upregulated *GPx1a* and *GPx1b* expression but did not affect *GPx4a* or *GPx4b* expression. The mechanism is still unclear and worth further study. Antioxidant enzymes are mediated by Nrf2 signaling, which protects cells from oxidative stress by binding to Keap1 [37]. In the present study, vitamin D upregulated the mRNA and protein levels of *Nrf2* in the nucleus and downregulated *Keap1a* (but not *Keap1b*), potentially contributing to the formation of phospholipids. Phospholipids can upregulate *Keap1a* (and not *Keap1b*) mRNA expression in fish intestines [38], and vitamin D has been shown to regulate phospholipid metabolism in human hepatocytes [39]. Therefore, we speculate that vitamin D may have increased phospholipid content, leading to the upregulation of *Keap1a* (rather than *Keap1b*) mRNA in the fish skin; however, further studies are necessary to validate this hypothesis.

#### 3.2.2. Vitamin D Supplementation Decreased Cell Apoptosis, Mediated by the p38MAPK and JNK Pathways

Oxidative stress can directly induce cell apoptosis [40,41]. Two major pathways have been implicated in the molecular mechanism of apoptosis, including death receptor and mitochondrial pathways [42]. The death receptor pathway (FasL/caspase-8/[caspase-3 and caspase-7]) and the mitochondrial pathway ([Bax, Bcl-2, and Mcl-1]/Apaf-1/caspase-9/[caspase-3 and caspase-7]) are mediated by p38MAPK and JNK signaling, respectively [43,44,45]. In the present study, vitamin D supplementation downregulated the expression of *FasL*, *Bax*, *Apaf-1*, *p38MAPK*, *JNK*, and *caspase-2, 3*, *7*, *8*, *9*, and upregulated *Bcl-2*, *IAP*, and *Mcl-1* in fish skin, indicating that vitamin D inhibited cell apoptosis during pathogen infection, in association with the p38MAPK and JNK pathways. Under the lipopolysaccharide challenge, dietary vitamin D reduced the apoptosis rate of macrophages in the head kidney in yellow catfish [46]. Furthermore, vitamin D decreased *caspase-3* and *BAX* expression and increased *Bcl-2* expression in the intestine of turbot under the lipopolysaccharide challenge [6]. In the intestine of yellow catfish, dietary vitamin D downregulated transcription levels of *caspase-3*, *caspase-9*, and *BAX* [13]. These findings indicate that vitamin D has diverse effects on apoptosis.

#### 3.2.3. Vitamin D Supplementation Enhanced Tight Junction Function via MLCK Inhibition

The tight junction, a paracellular pathway between epithelial and endothelial cells, forms a regulatory barrier and contains membrane proteins [47]. Tight junction proteins, especially claudin proteins such as occludin and Zo-1, play an important role in maintaining barrier function. Claudins are responsible for the paracellular barrier function of tight junctions and, in some cases, confer paracellular channel functions to paracellular tight junction barriers [47]. The present study shows that vitamin D increased the mRNA expression of barrier-forming tight junction proteins, including *ZO-1*, *ZO-2*, *occludin,* and *claudins*, suggesting that vitamin D enhanced the tight junction barrier function in fish skin. Dietary vitamin D promoted the expression of *claudin-1*, *claudin-2*, *claudin-5,* and *claudin-12* in the intestine of yellow catfish [13]. These results demonstrate that vitamin D could improve tight junction function. It has been reported that inhibition of MLCK expression can improve epithelial tight junction barrier function [48]. Vitamin D supplementation decreased *MLCK* levels, and *MLCK* expression was negatively correlated with tight junction in our study, suggesting that vitamin D positively affected tight junction function in the skin via *MLCK* inhibition.

### 3.3. Vitamin D Supplementation Improved Immune Barrier Function in Fish Skin

Defense molecules, such as lysozyme, complement components, and antimicrobial peptides, are essential for mediating the innate immune system and protecting the body from microbial invasion [49]. In the present study, vitamin D supplementation increased lysozyme and ACP activities and C3, IgM, and C4 levels in the skin of grass carp, indicating that dietary vitamin D improved immune function in fish skin. Immune system homeostasis is related to inflammatory cytokines, including pro-inflammatory cytokines, such as TNF-α and IL-6, and anti-inflammatory cytokines, such as IL-10 [50]. Excessive secretion of TNF-a and IL-6 further stimulates the secretion of other pro-inflammatory cytokines and aggravates damage [51]. IL-10 is a vital regulator in the maintenance of immunological homoeostasis [52]. In the present study, vitamin D supplementation downregulated the expression of pro-inflammatory cytokines (except for *IL-1β* and *-12p40*) and upregulated the expression of anti-inflammatory cytokines (except for *TGF-β2*), indicating that vitamin D affects inflammatory responses and supports normal immune function in fish skin. In trout (*Scophthalmus maximus* L.), optimal vitamin D supplement decreased the mRNA levels of *IL-1β*, *IL-6*, *IL-8,* and *TNF-α* in the liver and hindgut [53]. Under *Edwardsiella tarda* infection, vitamin D depressed *IL-17*, *IL-1β*, and *IL-6* expression in the distal intestine and spleen while upregulating *IgM* and *TGF-β* expression in the trout intestine [54]. These data show that vitamin D can inhibit the expression of pro-inflammatory cytokines and enhance an anti-inflammatory response.

Further studies are necessary to verify the effect of vitamin D on some inflammatory cytokines. For instance, vitamin D supplementation did not contribute to *IL-1β* or *IL-12p40* mRNA expression (rather than *IL-12p35*). Previous studies have shown that *IL-1β* enhanced *IL-12p40* expression in the Atlantic salmon (*Salmo salar*) but did not affect *IL-12p35* expression [55]. Therefore, vitamin D supplementation likely had no effects on *IL-1β* expression, which in turn did not change the *IL-12p40* mRNA levels. Additionally, vitamin D supplementation affected *TGF-β1* expression but did not affect *TGF-β2* expression, which could be attributed to methionine content. Vitamin D supplementation has been shown to enhance methionine content in the intestines of grass carp [10]; however, methionine dipeptide did not affect *TGF-β2* expression [56]. However, further studies are necessary to verify these hypotheses.

Pro-inflammatory cytokines are mediated by the *IKK*/*IκBα*/*NF-κB* pathway in mammals [57]. In the present study, vitamin D supplementation decreased *NF-κBp65* (but not *NF-κBp52*), *IKKβ*, and *-γ* expression at both the mRNA and protein levels but upregulated *IκBα* expression, indicating that vitamin D suppressed pro-inflammatory responses by decreasing *NF-κBp65* protein translocation to the nucleus in fish skin. Under *Edwardsiella ictalur*i infection, dietary vitamin D decreased the mRNA levels of *NF-κBp65* and *NF-κBp52* and increased those of *IκBα* in yellow catfish [58]. Notably, *NF-κBp52* and *IKKα* expression levels were not affected by vitamin D supplementation in our study. *NF-κB* signaling exerts its effects through canonical and non-canonical pathways. The former relies on *IκB* kinase activation (*IKKβ* and *γ*) and *IκBα* degradation, causing *NF-κBs* (*p65*/*p50*/*c-Rel*) to translocate into the nucleus. Conversely, the latter depends on *IKKα* activation, resulting in the formation of p52/RelB dimers [57]. In the present study, neither *IKKα* nor *NF-κBp52* mRNA levels were influenced by vitamin D supplementation, indicating that vitamin D-induced modulation of proinflammatory factors was probably related to *NF-κB* canonical signaling rather than the non-canonical signaling pathway. Additionally, *TOR* signaling is vital for regulating anti-inflammatory cytokines in fish [32]. In the present study, vitamin D supplementation downregulated *4E-BP1* (rather than *4E-BP2*) expression, upregulated *TOR* and *S6K1* expression, and increased p-TORser2448 protein levels, indicating that vitamin D-induced increase in anti-inflammatory cytokines in fish skin was partly associated with TOR signaling. However, vitamin D supplementation did not affect *4E-BP2* expression, which might be attributed to the phosphorylation of eukaryotic initiation factor 2 alpha (eIF2α). eIF2α phosphorylation has been shown to upregulate *4E-BP1* expression (not *4E-BP2*) in mice [59], and vitamin D has been shown to enhance eIF2α phosphorylation in endothelial cells [60]. However, further studies are necessary to verify this hypothesis.

### 3.4. Vitamin D Supplementation Improved VDR Expression in Fish Skin

Presently, one VDR gene has been identified in the mammalian genome, whereas two VDR genes (*VDRa* and *VDRb*) are present in fish [61,62]. Studies on the role of VDR isoforms in vitamin D-mediated immune response are limited. In the present study, vitamin D supplementation upregulated *VDRa* expression but did not affect *VDRb* transcription. Correlation analysis indicated that *VDRa* is associated with *Nrf2*, *MLCK*, *IκBα*, *IKKβ*, *IKKγ*, *c-Rel*, *TOR, S6K1,* and *4E-BP1* signaling molecules, indicating that VD/VDRa-mediated antioxidant capability, cell apoptosis, tight junction function, and immune response in the skin of fish were at least partly correlated with *Nrf2*, *MLCK*, *NF-κB*, and *TOR* signaling. Lin et al. [63] demonstrated that *VDRa* regulates epithelial calcium channels to increase calcium uptake. Moreover, *VDRb* (but not *VDRa*) loss has been shown to cause craniofacial cartilage malformation in zebrafish [64], indicating that *VDRa* and *VDRb* play different roles in fish. Under the lipopolysaccharide challenge, VDRa and VDRb participated in the depression of cell apoptosis and autophagy in trout intestine [6]. Furthermore, a previous study has demonstrated that VDR genes are universally expressed in fish, with a higher transcription level of *VDRa* than of *VDRb* [65]. Our unpublished data found that *VDRa* was highly expressed in the skin compared with *VDRb*. This observation supports our conclusion that *VDRa* plays pleiotropy roles in the skin mucosal barrier, and elucidation of its specific functions warrants further investigation.

## 4. Conclusions

In the present study, we examined the effects of vitamin D on physical and immune barrier functions in fish skin following *A. hydrophila* infection. Vitamin D improves physical barrier function in fish skin by promoting antioxidant capacity, decreasing excessive apoptosis, and enhancing tight junction barriers. Additionally, vitamin D supplementation improves immune barrier function by promoting disease resistance, increasing antibacterial compound and immunoglobulin production, downregulating pro-inflammatory cytokines, and upregulating anti-inflammatory cytokines. Moreover, vitamin D-induced improvement in the skin mucosal barrier system was mediated by NrF2, p38MAPK, JNK, MLCK, NF-κB, and TOR signaling pathways. VDRa was also shown to be activated by dietary vitamin D and involved in mucosal barrier regulation. Collectively, these data indicate that vitamin D enhances the skin mucosal barrier in fish under pathogen infection.

## 5. Materials and Methods

This study was approved by the relevant ethics board, and all procedures were conducted in accordance with the guidelines of the University of Sichuan Agricultural Animal Care Advisory Committee (Sichuan, China).

### 5.1. Experimental Diets and Conditions

The compositions of the experimental diets are shown in Supplementary Table S1. The vitamin D levels in the present study were chosen based on previous studies [5,66,67,68,69,70,71] (Supplementary Table S2). The experimental diets comprised one control diet (un-supplemented) and five diets supplemented with vitamin D_3_ (500,000 IU/kg) at 400, 800, 1200, 1600, and 2000 IU/kg feed. The final concentrations of vitamin D_3_ in each group were 15.2 (control; VD15.2), 364.8 (VD364.8), 782.5 (VD782.5), 1167.9 (VD1167.9), 1573.8 (VD1573.8), and 1980.1 (VD1980.1) IU/kg of feed, as verified by high-performance liquid chromatography [10]. The milled ingredients were mixed with oil and distilled water, extruded into pellets, and stored at −20 °C until use.

Healthy grass carp were procured from a local farm (Chengdu, Sichuan, China) and acclimatized for four weeks in a culturing fishpond, during which time they were fed basal diet (control) for two weeks to reduce vitamin D_3_ stores in the body. The fish selected for the experiments were visibly healthy, and no parasites were found under microscopic examination. Prior to the experiment, 540 individuals (257.24 ± 0.63 g, mean ± standard deviation (SD) were randomly distributed into 18 culture cages (1.4 × 1.4 × 1.4 cm; 30 fish per cage) and fed the respective diets for 70 d. The growth trial was performed under natural photoperiod, with daily monitoring of the water temperature (28.0 ± 2.1 °C), oxygen (>6 mg/L), and pH (7.0 ± 0.2).

### 5.2. Challenge Trial and Sampling

The challenge trial and sampling were performed according to established procedures [72]. After the growth trial, 15 fish with similar weights were randomly selected, fasted for 24 h, and intraperitoneally injected with 1 mL of *A. hydrophila* at a concentration of 2.5 × 10^8^ colony-forming units (CFU)/mL to examine the effects of vitamin D on barrier function. The fish were returned to their cages for two weeks and fed their respective diets during the infection period. The protocol for the challenge experiment was appropriately adjusted to induce inflammation and reactivity against the pathogen without causing mortality [73]. The experimental conditions during the challenge period were similar to those during the growth trial. At the end of the challenge trial, the fish were starved for 24 h, euthanized in a benzocaine bath (50.0 mg/L), and the skin was immediately extracted and frozen at −80 °C and 4% paraformaldehyde for further analyses.

### 5.3. Biochemical Analysis

Fish skin was homogenized with 10× sterile ice-cold physiological saline at 0.9% (*w*/*v*), and the supernatant was collected after centrifuging at 6000× *g* for 20 min at 4 °C. The methods for the analysis of oxidative damage biomarkers and antioxidant enzymes are described in Supplementary Table S3.

### 5.4. Hematoxylin and Eosin Staining of Fish Skin

The hematoxylin and eosin staining procedure was conducted according to established procedures [9]. The fish skin was dehydrated in gradually increasing concentrations of ethanol, cleared with xylene, and embedded in paraffin. The embedded samples were dissected into 4-μm slices and stained with hematoxylin and eosin. A Nikon TS100 light microscope (Nikon, Tokyo, Japan) was used to observe structural morphology.

### 5.5. Total RNA Extraction and Real-Time qPCR

RNA extraction, reverse transcription, and qPCR were performed according to the procedures described by Sun et al. [74]. Total RNA was extracted from the skin samples using the RNAiso Plus kit (Takara, Dalian, China), according to the manufacturer’s instructions, and treated with DNAse I (D7073, Beyotime, Shanghai, China). RNA quality and quantity were determined using 1% agarose gel electrophoresis and Nanodrop 2000 spectrophotometer (Thermo Scientific, Waltham, MA, USA), respectively. RNA was reverse-transcribed to generate cDNA using a PrimeScript™ RT Reagent Kit (Takara, Dalian, China). qPCR was performed on a Real-Time System (QuanStudio 5, Life Technologies, Carlsbad, CA, USA) using a reaction mixture containing 1 μL of cDNA, 0.2 μL of ROX, 5 μL of SYBR Premix Ex Taq, 0.4 μL of primers, and 3.4 μL of nuclease-free water (Vazyme, Nanjing, China). The relative gene expression was normalized to that of *β-actin* (internal control) and calculated using the 2^−ΔΔCT^ method, according to Livak and Schmittgen [75]. The specific primers used for qPCR are listed in Supplementary Table S4.

### 5.6. Western Blot Analysis

Western blot analysis, including skin homogenate preparation and primary and secondary antibody selection, were performed following previously described procedures [76]. Briefly, the skin sample was ground into powder, homogenized, extracted in protein lysis solution (RIPA: PMSF = 80:1) for 30 min, and centrifuged at 6000× *g* for 15 min at 4 °C. The tissue supernatant was exacted, treated with 5× loading buffer (Beyotime Biotechnology Inc., Nanjing, China), and analyzed using a BCA assay kit (Beyotime Biotechnology Inc.). Subsequently, the proteins were loaded onto a sodium dodecyl sulfate–glycine polyacrylamide gel and transferred onto a polyvinylidene fluoride membrane. The membranes were blocked by incubating in bovine serum albumin (5%) for 2 h, followed by incubation with primary antibodies overnight at 4 °C. Thereafter, the polyvinylidene fluoride membranes were incubated with secondary antibodies for 2 h, and protein signals were visualized using the ChemiDoc imaging system (Bio-Rad, Hercules, CA, USA). Protein levels were quantified using the ImageJ software (NIH, Bethesda, NJ, USA; 1.42q). Information on the antibodies is included in Supplementary Table S5.

### 5.7. Statistical Analysis

Data were presented as mean ± SD. All results were normalized using the Shapiro–Wilk test and Levene’s test was used for variance homogeneity testing before data analysis. Statistical differences between means were determined using a one-way analysis of variance, followed by Tukey’s multiple range test, and means were considered significant at *p* < 0.05. All statistical analyses were performed using SAS PROC MIXED software version 9.4 (SAS Institute, Inc., Cary, NC, USA) as described previously [77,78]. Orthogonal polynomial contrasts were used to assay the linear and quadratic effects of increasing vitamin D concentration. Data visualization was performed using GraphPad 8.0 (GraphPad Software, Inc., La Jolla, CA, USA).

## Data Availability

All datasets generated for this study are included in the manuscript and/or the Supplementary Files.

## Supplementary Materials

The following supporting information can be downloaded at: https://www.mdpi.com/article/10.3390/ijms241411243/s1.

## Abbreviations

4E-BP, 4E-binding protein 1; ACP, acid phosphatase; AHR, anti-hydroxy radical; Apaf1, apoptotic protease activating factor-1; ASA, anti-superoxide anion; BAX, BCL2-associated X; Bcl-2, B-cell lymphoma-2; Caspase, cysteinyl aspartate-specific proteinase; CAT, catalase; C3, complement 3; FasL, ligands associated with apoptosis; GPx, glutathione peroxidase; GSH, glutathione; GST, glutathione reductase; GR, glutathione reductase; IAP, inhibitor of apoptosis protein; IgM, immunoglobulin M; IFN, interferon; IL, interleukin; IκBα, inhibitor of NF-κB; IKK, inhibitor of kappa B kinase; JNK, c-Jun N-terminal kinase; Keap1, kelch-like ECH-associated protein 1; MAPK, mitogen-activated protein kinase; Mcl-1, myeloid cell leukemia-1; MDA, malondialdehyde; MLCK, myosin light-chain kinase; NF-κB, nuclear factor kappa B; Nrf2, nuclear factor E2-related factor 2; PC, protein carbonyl; p38MAPK, mitogen-activated protein kinases; ROS, reactive oxygen species; S6K1, ribosomal protein S6 kinase 1; SOD, superoxide dismutase; T-AOC, Total antioxidant capacity; TGF, transforming growth factor; TNF, tumor necrosis factor; TOR, target of rapamycin; VDR, vitamin D receptor. ZO-1, zonula occludens-1.
