# Supplementary material for: Vitamin D Promotes Mucosal Barrier System of Fish Skin Infected with Aeromonas hydrophila through Multiple Modulation of Physical and Immune Protective Capacity
Source: Int J Mol Sci. 2023 Jul 8;24(14):11243. doi: 10.3390/ijms241411243 (PMC10379486; doi:10.3390/ijms241411243)
Supplement: Supplementary file 1 [file ijms-24-11243-s001.zip › ijms-2340879-supplementary.pdf]

Supplementary Material

Vitamin D promotes mucosal barrier system of fish skin infected with *Aeromonas hydrophila* through multiple modulation of physical and immune protective capacity

Supplementary Tables

Supplementary Table S1. Composition and nutrient levels of basal diet.

Ingredients (g/kg)		Nutrients content (g/kg) [1]	
Casein	135.0	Crude protein	300.0
Gelatin	44.7	Crude fat	37.8
Soybean protein concentrate	220.0	n-3 PUFAs[5]	10.4
α-starch	240.0	n-6 PUFAs	9.6
Corn starch	209.1	n-3: n-6 PUFAs	10.8
linseed oil	17.7	Available phosphorus	4.0
Soy oil	14.5	Vitamin D_3 (ug/kg)	3.8
Cellulose	50.0		
$Ca(H_2PO_4)_2$	14.8		
Vitamin premix [2]	10.0		
Mineral premix [3]	20.0		
Vitamin D_3 premix [4]	10.0		
Choline chloride (50%)	10.0		
Ethoxyquin (30%)	0.5		
DL-Met	3.7		
In total	1000.0		

[1] The contents of Crude protein and lipid contents were measured on an air-dried matter basis. Supplementation of available P, n-3 PUFAs, and n-6 PUFAs was calculated according to NRC (2011).

[2] Per kg of vitamin premix, g/kg: DL -α-tocopherol acetate (50%), 12.58; menadione (22·9%), 0.83; cyanocobalamin (1%), 0.94; D-biotin (2%), 0.75; folic acid (95%), 0.42; thiamine nitrate (98%), 0.09; ascorbyl acetate (95%), 4.31; niacin (99%), 4.04; meso-inositol (98%), 19.39; calcium-D-pantothenate (98%), 3.85; riboflavin (80%), 0.73; pyridoxine hydrochloride (98%), 0.62; retinyl acetate (500,000 IU/g), 2.10. All ingredients were diluted with 1 kg maize starch.

[3] Per kg of mineral premix (g/kg): $FeSO_4.H_2O$ (30.0% Fe), 12.2500; $MgSO_4.H_2O$ (15.0% Mg), 200.0000; $ZnSO_4.H_2O$ (34.5% Zn), 8.2460; $MnSO_4.H_2O$ (31.8% Mn), 2.6590; $CuSO_4.5H_2O$ (25.0% Cu), 0.9560; Na_2SeO_3 (44.7% Se), 0.0168; KI (76.9% I), 0.0650 g. All ingredients were diluted with maize starch to 1 kg.

[4] Vitamin D_3 premix: premix was added to obtain graded level of vitamin D_3 and the amount of maize starch was reduced to compensate.

[5]PUFAs = polyunsaturated fatty acids

Supplementary Table S2. Vitamin D requirements of different fish species[1]

Feeding habits	Species	Vitamin D levels (μg/kg)	Requirement (μg/kg)
Herbivores	Juvenile grass carp (*Ctenopharyngodon idella*) [66]	0, 2.5, 5, 12.5, 25, 50, 75	25
	Wuchang bream (*Megalobrama amblycephala*) [67]	0, 12.5, 25, 50, 100, 200	100
Omnivores	Siberian sturgeon (*Acipenser baerii*) [68]	1.5, 6, 11.25, 22, 41.75, 82.5, 2500	41.75
	Hybrid tilapia (*Oreochromis niloticus*×*O.aureus*) [69]	0, 7.5, 15, 22.5, 30, 37.5, 45, 52.5	9.37
Predators	European sea bass (*Dicentrachus labrax*) [70]	0, 480, 960, 3500	480
	Asian swamp eel (*Monopterus albus*) [71]	0, 6.25, 12.5, 25, 50, 100	12.5
	Atlantic salmon (*Salmo salar*) [5]	200, 5000, 57000	5000

[1] References [5, 66-71] are cited in the main text.

Supplementary Table S3. Method of analysis of biomarker and enzymes activity-related parameters

Indices	Analysis method
Reactive oxygen species (ROS)	Chemical fluorescence method (Biyuntian, Shanghai, China).
Malondialdehyde (MDA)	The thiobarbituric acid (TBA) assay kit (Nanjing Jiancheng Institute, China)
Protein carbonyl (PC)	The 2, 4-dinitrophenylhydrazine (DNPH) reagent (Nanjing Jiancheng Institute, China)
Anti-superoxide anion (ASA)	The superoxide anion free radical detection Kit (Nanjing Jiancheng Institute, China)
Anti-hydroxy radical (AHR)	The hydroxyl free radical detection Kit (Nanjing Jiancheng Institute, China)
Superoxide dismutase (SOD)	The Hydroxylamine method (Nanjing Jiancheng Institute, China)
Catalase (CAT)	Visible light, commercial kit (Nanjing Jiancheng Institute, China)
Glutathione peroxidase (GPx)	Colorimetric method, commercial kit (Nanjing Jiancheng Institute, China)
Glutathione-S-transferase (GST)	Colorimetric method, commercial kit (Nanjing Jiancheng Institute, China)
Glutathione reductase (GR)	A commercial kit (Nanjing Jiancheng Institute, China)
Glutathione (GSH)	Spectrophotometric method, commercial kit (Nanjing Jiancheng Institute, China)
Protein concentrations	Coomassie brilliant blue method, commercial kit (Nanjing Jiancheng Institute, China)
Lysozyme	Spectrophotometry, commercial kit (Nanjing Jiancheng Institute, China)
Acid phosphatase (ACP)	Spectrophotometry, commercial kit (Nanjing Jiancheng Institute, China)
Complement (C3)	The immunoturbidimetry kit (Yilikang Biotechnology co., Ltd., Zhejiang, China)
Complement (C4)	The immunoturbidimetry kit (Yilikang Biotechnology co., Ltd., Zhejiang, China)
Immunoglobulin (IgM)	The immunoturbidimetry kit (Yilikang Biotechnology co., Ltd., Zhejiang, China)

Supplementary Table S4. Real-time PCR primer sequences[1].

Target Gene	Primer sequence Forward (5' → 3')	Primer sequence Reverse (5' → 3')	Temperature(°C)	Accession number
CuZnSOD	CGCACTTCAACCCTTACA	ACTTTCCTCATTGCCTCC	61.5	GU901214
MnSOD	ACGACCCAAGTCTCCCTA	ACCCTGTGGTTCTCCTCC	60.4	GU218534
CAT	GAAGTTCTACACCGATGAGG	CCAGAAATCCCAAACCAT	58.7	FJ560431
GPx1a	GGGCTGGTTATTCTGGGC	AGGCGATGTCATTCCTGTTC	61.5	EU828796
GPx1b	TTTTGTCCTTGAAGTATGTCCGTC	GGGTCGTTCATAAAGGGCATT	60.3	KT757315
GPx4a	TACGCTGAGAGAGGTTTACACAT	CTTTTCCATTGGGTTGTTCC	60.4	KU255598
GPx4b	CTGGAGAAATACAGGGGTTACG	CTCCTGCTTTCCGAACTGGT	60.3	KU255599
GSTR	TCTCAAGGAACCCGTCTG	CCAAGTATCCGTCCCACA	58.4	EU107283
GSTp1	ACAGTTGCCCAAGTTCCAG	CCTCACAGTCGTTTTTTCCA	59.3	KM112099
GSTp2	TGCCTTGAAGATTATGCTGG	GCTGGCTTTTATTTCACCCT	59.3	KP125490
GSTo1	GGTGCTCAATGCCAAGGGAA	CTCAAACGGGTCGGATGGAA	58.4	KT757314
GSTo2	CTGCTCCCATCAGACCCATTT	TCTCCCCTTTTCTTGCCCATA	61.4	KU245630
GR	GTGTCCAACTTCTCCTGTG	ACTCTGGGGTCCAAAACG	59.4	JX854448
Nrf2	CTGGACGAGGAGACTGGA	ATCTGTGGTAGGTGGAAC	62.5	KF733814
Keap1a	TTCCACGCCCTCCTCAA	TGTACCCTCCCGCTATG	63.0	KF811013
Keap1b	TCTGCTGTATGCGGTGGGC	CTCCTCCATTCATCTTTCTCG	57.9	KJ729125
FasL	AGGAAATGCCCGCACAAATG	AACCGCTTTCATTGACCTGGAG	61.4	KT445873
p38MAPK	TGGGAGCAGACCTCAACAAT	TACCATCGGGTGGCAACATA	60.4	KM112098
JNK	ACAGCGTAGATGTGGGTGATT	GCTCAAGGTTGTGGTCATACG	62.3	KT757312
Bcl-2	AGGAAAATGGAGGTTGGGAT	CTGAGCAAAAAAGGCGATG	60.3	JQ713862
Mcl-1	TGGAAAGTCTCGTGGTAAAGCA	ATCGCTGAAGATTTCTGTTGCC	58.4	KT757307
Bax	CATCTATGAGCGGGTTCGTC	TTTATGGCTGGGGTCACACA	60.3	JQ793788
Apaf-1	AAGTTCTGGAGCCTGGACAC	AACTCAAGACCCCACAGCAC	61.4	KM279717

IAP	CACAATCCTGGTATGCGTCG	GGGTAATGCCTCTGGTGCTC	58.4	FJ593503.1
Caspase-2	CGCTGTTGTGTGTTTACTGTCTCA	ACGCCATTATCCATCTCCTCTC	60.3	KT757313
Caspase-3	GCTGTGCTTCATTTGTTTG	TCTGAGATGTTATGGCTGTC	55.9	JQ793789
Caspase-7	GCCATTACAGGATTGTTTCACC	CCTTATCTGTGCCATTGCGT	57.1	KT625601
Caspase-8	ATCTGGTTGAAATCCGTGAA	TCCATCTGATGCCCATACAC	59.0	KM016991
Caspase-9	CTGTGGCGGAGGTGAGAA	GTGCTGGAGGACATGGGAAT	59.0	JQ793787
Occludin	TATCTGTATCACTACTGCGTCG	CATTCACCCAATCCTCCA	59.4	KF193855
ZO-1	CGGTGTCTTCGTAGTCGG	CAGTTGGTTTGGGTTTCAG	59.4	KJ000055
ZO-2	TACAGCGGGACTCTAAAATGG	TCACACGGTCGTTCTCAAAG	60.3	KM112095
Claudin-b	GAGGGAATCTGGATGAGC	ATGGCAATGATGGTGAGA	57.0	KF193860
Claudin-c	GAGGGAATCTGGATGAGC	CTGTTATGAAAGCGGCAC	59.4	KF193859
Claudin-f	GCTGGAGTTGCCTGTCTTATTC	ACCAATCTCCCTCTTTTGTGTC	57.1	KM112097
Claudin-3c	ATCACTCGGGACTTCTA	CAGCAAACCCAATGTAG	57.0	KF193858
Claudin-7a	ACTTACCAGGGACTGTGGATGT	CACTATCATCAAAGCACGGGT	59.3	KT625604
Claudin-7b	CTAACTGTGGTGGTGATGAC	AACAATGCTACAAAGGGCTG	59.3	KT445866
Claudin-11	TCTCAACTGCTCTGTATCACTGC	TTTCTGGTTCACTTCCGAGG	62.3	KT445867
Claudin-12	CCCTGAAGTGCCCACAA	GCGTATGTCACGGGAGAA	55.4	KF998571
Claudin-15a	TGCTTTATTTCTTGGCTTTC	CTCGTACAGGGTTGAGGTG	59.0	KF193857
Claudin-15b	AGTGTTCTAAGATAGGAGGGGAG	AGCCCTTCTCCGATTTCAT	62.3	KT757304
MLCK	GAAGGTCAGGGCATCTCA	GGGTCGGGCTTATCTACT	53.0	KM279719
TGF-β1	TTGGGACTTGTGCTCTAT	AGTTCTGCTGGGATGTTT	55.9	EU099588
TGF-β2	TACATTGACAGCAAGGTGGTG	TCTTGTTGGGGATGATGTAGTT	55.9	KM279716
IL-4/13A	CTACTGCTCGCTTTCGCTGT	CCCAGTTTTCAGTTCTCTCAGG	55.9	KT445871
IL-4/13B	TGTGAACCAGACCCTACATAACC	TTCAGGACCTTTGCTGCTTG	55.9	KT625600

IL-10	AATCCCTTTGATTTTGCC	GTGCCTTATCCTACAGTATGTG	61.4	HQ388294
IL-11	GGTTCAAGTCTCTTCCAGCGAT	TGCGTGTTATTTTGTTCAGCCA	57.0	KT445870
TNF-α	CGCTGCTGTCTGCTTCAC	CCTGGTCCTGGTTCACTC	58.4	HQ696609
IFN-γ2	TGTTTGATGACTTTGGGATG	TCAGGACCCGCAGGAAGAC	60.4	JX657682
IL-1β	AGAGTTTGGTGAAGAAGAGG	TTATTGTGGTTACGCTGGA	57.1	JQ692172
IL-6	CAGCAGAATGGGGGAGTTATC	CTCGCAGAGTCTTGACATCCTT	62.3	KC535507.1
IL-8	ATGAGTCTTAGAGGTCTGGGT	ACAGTGAGGGCTAGGAGGG	60.3	JN663841
IL-12p35	TGGAAAAGGAGGGGAAGATG	AGACGGACGCTGTGTGAGTGTA	55.4	KF944667.1
IL-12p40	ACAAAGATGAAAAACTGGAGGC	GTGTGTGGTTTAGGTAGGAGCC	59.0	KF944668.1
IL-15	CCTTCCAACAATCTCGCTTC	AACACATCTTCCAGTTCTCCTT	61.4	KT445872
IL-17D	GTGTCCAGGAGAGCACCAAG	GCGAGAGGCTGAGGAAGTTT	62.3	KF245426.1
VDRa	CAGAGAGTCGTACCGTTTCG	ACAAGTCTTTGCCTTTCAGC	62.5	MW789222
VDRb	ATTGACACGCTGGTGGATGC	CTGGAGAGTGGCTGAAGGAGTC	64.0	MW789223
NF-κBp65	GAAGAAGGATGTGGGAGATG	TGTTGTCGTAGATGGGCTGAG	62.3	KJ526214
NF-κBp52	TCAGTGTAACGACAACGGGAT	ATACTTCAGCCACACCTCTCTTAG	58.4	KM279720
c-Rel	GCGTCTATGCTTCCAGATTTACC	ACTGCCACTGTTCTTGTTCACC	59.3	KT445865
IκBα	TCTTGCCATTATTCACGAGG	TGTTACCACAGTCATCCACCA	62.3	KJ125069
IKKα	GGCTACGCCAAAGACCTG	CGGACCTCGCCATTCATA	60.3	KM279718
IKKβ	GTGGCGGTGGATTATTGG	GCACGGGTTGCCAGTTTG	60.3	KP125491
IKKγ	AGAGGCTCGTCATAGTGG	CTGTGATTGGCTTGCTTT	58.4	KM079079
β-actin	GGCTGTGCTGTCCCTGTA	GGGCATAACCCTCGTAGAT	61.4	M25013

[1] SOD, superoxide dismutase; CAT, catalase; GPx, glutathione peroxidase; GST, glutathione S-transferase; GR, glutathione reductase; Nrf2, NF-E2-related factor 2; FasL, ligands associated with apoptosis; MAPK, mitogen-activated protein kinase; JNK, cJ N-terminal kinase; Bcl-2, B-cell lymphoma-2; Mcl-1, myeloid cell leukemia-1; BAX, BCL2-associated X;Apaf1, apoptotic protease activating factor-1; IAP, inhibitor of apoptosis protein; caspase, cysteinyl aspartate specific proteinase; MLCK, myosin light chain kinase; TGF-

β, transforming growth factor β; TNF-α, tumor necrosis factor α; IFN-γ2, interferon γ2 IL, interleukin; VDR, vitamin D receptor; NF-κB, nuclear factor kappa B; IκBα, inhibitor of kBα; IKK, IκB kinase.

Supplementary Table S5. Western blot staining antibodies

Indices	Host	Source	Catalog No.	Dilution
Nucleus Nrf2	Rabbit	Abcam (Cambridge, MA, USA))	ab31163	1:1000
β-actin	Rabbit	Affinity (Golden, Colorado, USA)	AF7018	1:3000
Lamin B1	Rabbit	Affinity (Golden, Colorado, USA)	AF5161	1:1000
MLCK	Rabbit	Affinity (Golden, Colorado, USA)	AF5314	1:1000
P38MAPK	Rabbit	Abclonal (Wuhan, Hubei,CN)	A14401	1:900
JNK	Rabbit	Abclonal (Wuhan, Hubei,CN)	A0288	1:1000
Total TOR	Rabbit	Affinity (Golden, Colorado, USA)	AF6308	1:1000
p-TOR Ser 2448	Rabbit	Affinity (Golden, Colorado, USA)	AF3308	1:1000
Anti-NF-κB p65	Rabbit	Affinity (Golden, Colorado, USA)	AF5006	1:750
VDR	Rabbit	Abclonal (Wuhan, Hubei,CN)	A2194	1:1200
